# Ultrasmall Coordination Polymers for Alleviating ROS-Mediated Inflammatory and Realizing Neuroprotection against Parkinson's Disease

**DOI:** 10.34133/2022/9781323

**Published:** 2022-07-18

**Authors:** Guowang Cheng, Xueliang Liu, Yujing Liu, Yao Liu, Rui Ma, Jingshan Luo, Xinyi Zhou, Zhenfeng Wu, Zhuang Liu, Tongkai Chen, Yu Yang

**Affiliations:** ^1^Key Laboratory of Modern Preparation of Traditional Chinese Medicine, Ministry of Education, Jiangxi University of Chinese Medicine, Nanchang 330004, China; ^2^Institute of Molecular Medicine (IMM), Renji Hospital, School of Medicine, Shanghai Jiao Tong University, Shanghai 200240, China; ^3^Science and Technology Innovation Center, Guangzhou University of Chinese Medicine, Guangzhou 510405, China; ^4^School of Cellular and Molecular Medicine, University of Bristol, Bristol BS8 1TH, UK; ^5^Institute of Functional Nano & Soft Materials Laboratory (FUNSOM), Soochow University, Suzhou, Jiangsu 215123, China

## Abstract

Parkinson's disease (PD) is the second most common neurodegenerative disease globally, and there is currently no effective treatment for this condition. Excessive accumulation of reactive oxygen species (ROS) and neuroinflammation are major contributors to PD pathogenesis. Herein, ultrasmall nanoscale coordination polymers (NCPs) coordinated by ferric ions and natural product curcumin (Cur) were exploited, showing efficient neuroprotection by scavenging excessive radicals and suppressing neuroinflammation. In a 1-methyl-4-phenyl-1,2,3,6-tetrahydropyridine (MPTP)-induced mouse PD model, such ultrasmall Fe-Cur NCPs with prolonged blood circulation and BBB traversing capability could effectively alleviate oxidative stress, mitochondrial dysfunction, and inflammatory condition in the midbrain and striatum to reduce PD symptoms. Thus, this study puts forth a unique type of therapeutics-based NCPs that could be used for safe and efficient treatment of PD with potential in clinical translation.

## 1. Introduction

Parkinson's disease (PD), the second most common neurodegenerative disease in the world, is clinically characterized by dyskinesia, cognitive impairment, autonomic dysfunction, and other nonmotor impairments [1]. Global population aging is expected to increase the prevalence of PD and challenge medical and socio-economic care systems [2]. Over the last two decades, various treatment strategies (e.g., surgery, exercise, and pharmacotherapy) have been exploited to treat the PD. These strategies might delay the deterioration of motor disorders and reduce the morbidity and mortality but fail to completely halt disease progression [3]. Therefore, development of more advanced strategies that allow the inhibition of PD progression is urgently required.

Reactive oxygen species (ROS) are elevated during PD pathogenesis owing to the dysregulated dopamine metabolism, mitochondrial dysfunction, aging, and abnormal level of calcium, ferric, and glutathione [4, 5]. Excessive ROS, in turn, can induce oxidative stress and facilitate the aberrant accumulation of alpha-synuclein, thereby promoting the M1 polarization phenotype in microglia, eventually leading to neuroinflammation and the death of dopaminergic neurons [6–8]. Therefore, therapeutics with ROS scavenging properties could inhibit the development of PD by suppressing inflammatory conditions in the brain. However, the blood-brain barrier (BBB), formed by endothelial cells interacting with pericytes, astrocytes, neurons, and microglia in neurovascular units, limits the delivery of therapeutics into the central nervous system for the treatment of PD [9, 10]. Therefore, development of anti-PD medicine simultaneously capable of scavenging excessive radicals, suppressing neuroinflammation and efficiently crossing the BBB, is extremely in demand yet remains challenging.

Nanoscale coordination polymers (NCPs) and metal-organic frameworks (MOFs) are porous materials composed of metal ions connected by organic linkers and have garnered considerable interest in recent years. MOFs/NCPs are widely employed as drug delivery systems due to their vast surface areas, tailorable structure, tunable sizes, and biodegradability [11–13]. Although most of the developed MOFs/NCPs based nanomedicines have been focused on their features as nanocarriers to deliver imaging and therapeutic cargoes [14, 15], utilizing their inherent functions of the component unit of frameworks is largely ignored, thereby limiting on-demand design and fabrication of MOFs/NCPs for biomedical applications. Recently, we and others have employed therapeutics (e.g., cisplatin, photosensitizer, or photothermal agent) as organic linkers to construct MOFs/NCPs for cancer therapy [16–21]. However, to our best knowledge, no study has reported utilizing therapeutics-based MOFs/NCPs for neurodegenerative disease treatment.

**Figure 1:**
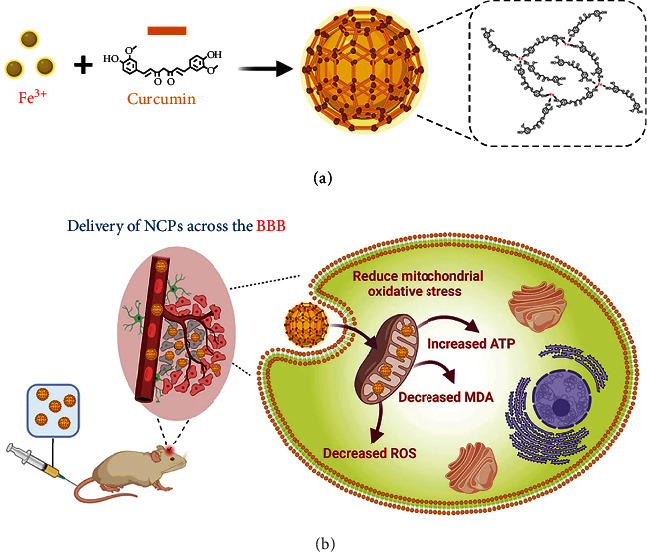
Schematic illustration of the preparation of Fe-Cur NCPs (a) and its anti-Parkinsonian effects (b).

Curcumin (Cur), the main constituent of turmeric, possesses significant anti-inflammatory capabilities and has been shown to alleviate Parkinson's disease [22–25]. With the symmetric phenolic hydroxyl groups of Cur, the drug-based ultrasmall NCPs, namely, Fe-Cur NCPs composed of ferric ions and Cur, were fabricated for anti-PD therapy (Figure 1). Specifically, the ultrasmall NCPs (several nanometers) showed excellent water dispersibility, prolonged blood circulation, and enhanced BBB crossing ability, thus significantly improving the therapeutics concentration in the brain. Furthermore, after coordination with ferric ions, such Fe-cur NCPs as Fe-based nanozymes endow multiple enzymes (e.g., catalase, superoxide dismutase)-like activities, significantly enhancing the capacities of scavenging excessive radicals. Both *in vitro* and *in vivo* experiments showed that the Fe-Cur NCPs could alleviate the mitochondrial dysfunction and improve the levels of DA and its metabolites in the midbrain and striatum. Moreover, the examination of brain glucose metabolism and immunofluorescence assays also showed that the Fe-Cur NCPs could repair the damage caused to dopaminergic neurons. Thus, Fe-Cur NCPs were validated as a potentially promising alternative strategy for ROS scavenging-based PD treatment.

## 2. Results and Discussions

### 2.1. Preparation and Characterization of Fe-Cur NCPs

**Figure 2:**
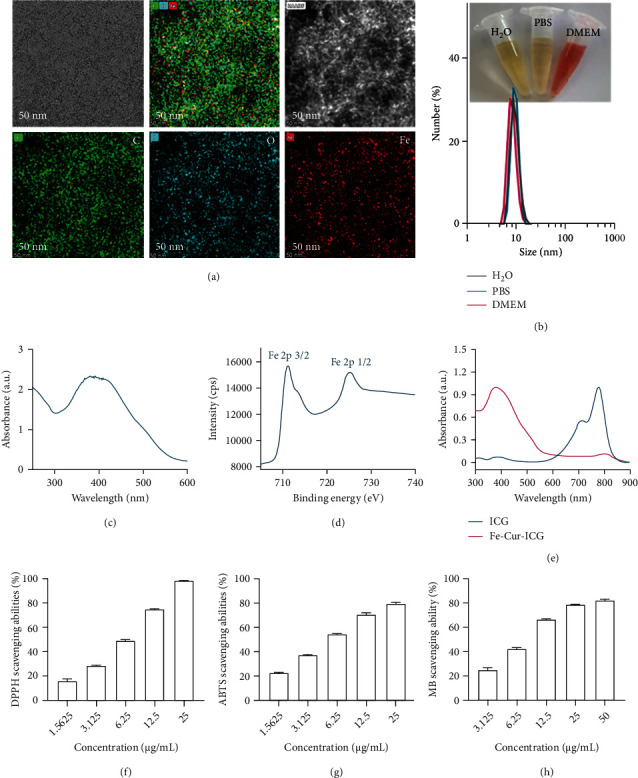
Characterization and ROS scavenging ability of the Fe-Cur NCPs. (a) TEM and corresponding TEM elemental mapping images of the Fe-Cur NCPs. (b) Sizes of Fe-Cur NCPs dispersed in H_2_O, PBS, and DMEM measured using dynamic light scattering (DLS). (c) UV-vis absorbance spectra of the Fe-Cur NCPs. (d) XPS spectra of Fe in Fe-Cur NCPs. (e) UV-vis absorbance spectra of free ICG and Fe-Cur-ICG NCPs. (f) DPPH radical scavenging ability of Fe-Cur NCPs at various concentrations. (g) ABTS scavenging ability of Fe-Cur NCPs at various concentrations. (h) ROS scavenging ability based on the absorbance of methylene blue after treatment with various concentrations of Fe-Cur NCPs (*n* =3).

Ultrasmall Fe-Cur NCPs (~10 nm) were prepared by a simple and efficient method by adding ferric ions into methanol solution containing curcumin, a nature product. Poly(vinylpyrrolidone) (PVP) was added to improve the water dispersity. After ferric ions were coordinated with the phenol groups of curcumin, the solution color changed from yellow to deep dark. Next, through dialysis against deionized water to remove excessive ferric ions and curcumin, ultrasmall Fe-Cur NCPs with the size about 10 nm were observed under transmission electron microscopy (TEM) (Figure 2(a)). At the same time, TEM elemental mappings (Figure 2(a)) revealed the distribution of C, O, and Fe elements in the Fe-Cur NCPs, further verifying the successful synthesis of the Fe-Cur NCPs. Dynamic light scattering (DLS) assays indicated that the hydrodynamic size of these NCPs was also about 10 nm, consistent with the sizes observed under TEM (Figure 2(b)). Meanwhile, the DLS also showed no significant change in particle size when the Fe-Cur NCPs were dispersed in phosphate-buffered saline (PBS) and cell culture medium (Dulbecco's Modified Eagle Medium, DMEM), indicating the excellent stability. Furthermore, the colloid stability of Fe-Cur NCPs in fetal bovine serum (FBS) at 37°C and in pH 7.4 PBS at 4°C was investigated. The results showed no obvious changes of the particle size and zeta potential, which indicated that Fe-Cur NCPs could maintain relatively good physiological stability in FBS at 37°C for 24 h, and in PBS at 4°C for 7 days (Figure S1). The excellent colloid stability is attributed to PVP, which acts as a dispersant and stabilizer in the synthesis process. Fe-Cur NCPs had amorphous structure as measured by X-ray diffraction (XRD) and showed the characteristic UV−vis absorption peak of Cur (~400 nm) (Figure S2, Figure 2(c)). Next, X-ray photoelectron spectroscopy (XPS) showed that the two strong binding energy peaks of 711 eV and 724 eV in the Fe-Cur NCPs were attributed to the Fe 2P_3/2_ and 2p_1/2_ of Fe (III), indicating the oxidation status of ferric ions in the obtained Fe-Cur NCPs (Figure S3, Figure 2(d)). To facilitate *in vivo* track of NCPs, we labelled the Fe-Cur NCPs with indocyanine green (ICG), which showed a characteristic UV-vis absorption peak at 780 nm (Figure 2(e)). Additionally, we also labelled the Fe-Cur NCPs with Fluorescein Isothiocyanate (FITC) convenient for *in vitro* imaging. Nanoscale coordination polymers (NCPs) and metal-organic frameworks (MOFs) have been widely used to load ICG and FITC according to previous works [18, 26]. The high labeling efficiency of ICG or FITC is attributed to the porous structure and coordination interaction of the NCPs and MOFs. To ensure whether the fluorescence labelled influence cellular uptake of Fe-Cur NCPs, we compared the particle size, zeta potential, and PDI of Fe-Cur-FITC NCPs and Fe-Cur-ICG NCPs with Fe-Cur NCPs. The results of the DLS showed that there were no obvious differences of the particle size, PDI, and zeta potential between Fe-Cur-FITC NCPs or Fe-Cur-ICG NCPs with Fe-Cur NCPs (Figure S4), indicating that fluorescent labeling to the Fe-Cur NCPs does not affect their function.

Next, three different types of free radical (e.g., 1, 1-Diphenyl-2-picrylhydrazyl (DPPH) radicals, 2,2′-azide (3-ethylbenzothiazoline-6-sulfonic acid) (ABTS), and •OH radicals) scavenging probes were adopted to determine the antioxidative ability of Fe-Cur-NCPs. For instances, DPPH radicals have a characteristic absorption peak at 517 nm, which would gradually disappear after adding antioxidants. We observed that the DPPH scavenging ability of Fe-Cur NCPs gradually increased as the increase of NCPs concentrations. At an NCP concentration of 25 *μ*g/mL (in terms of Fe elements), the DPPH scavenging ability was almost 100% (Figure 2(f)). In addition to DPPH, Fe-Cur NCPs also showed the same concentration-dependent ROS scavenging effects for ABTS and •OH radicals (Figure S5, Figures 2(g) and 2(h)). The above observation indicated that Fe-cur NCPs may show catalase-like and superoxide dismutase-like activities to efficiently scavenge excessive radicals. Importantly, Fe-Cur NCPs are assembled by the coordination interaction of curcumin and ferric ions, and this process does not affect the valence change of ferric ions. In addition, the XPS results in Figure S3 and Figure 2(d) showed that the two strong binding energy peaks of 711 eV and 724 eV in the Fe-Cur NCPs were attributed to the Fe 2p_3/2_ and 2p_1/2_ of Fe (III), indicating the curcumin could not reduce the ferric ions to ferrous nor attenuate the antioxidation efficacy of the Fe-Cur NCPs.

### 2.2. BBB Transport Evaluation *In Vitro*

**Figure 3:**
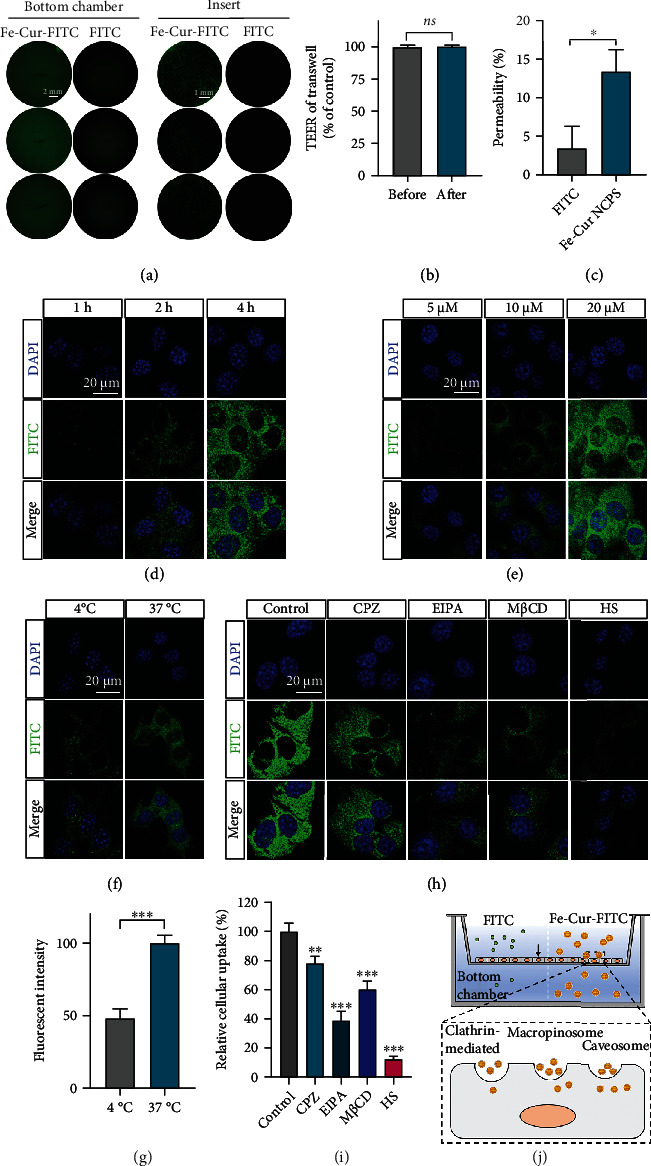
*In vitro* BBB transport evaluation. (a) Fluorescence images of the bottom chamber and insert part in the Transwell after 2 h incubation of FITC and Fe-Cur-FITC NCPs, respectively. Scale bar: bottom chamber 2 mm, insert 1 mm. (b) The TEER of bEnd.3 monolayer before and after incubation of Fe-Cur-FITC NCPs. (c) Permeability of FITC and Fe-Cur-FITC NCPs in the Transwell. Time-dependent (d) and concentration-dependent (e) cellular uptake of Fe-Cur-FITC in bEnd.3 cells. Scale bar 20 *μ*m. (f) Fluorescence images of b.End. 3 cells incubated with Fe-Cur-FITC at various temperatures. Scale bar: 20 *μ*m. (g) Quantitative analysis of fluorescent intensities based on fluorescence images (f). (h) Cellular uptake of Fe-Cur-FITC by bEnd.3 cells after treatment with different uptake inhibitor. Scale bar: 20 *μ*m. (i) Quantification results of cellular uptake (h). (j) Schematic illustration of Fe-Cur-FITC transporting the *in vitro* BBB model and its mechanism. *P* values were calculated by Tukey s post-test (^∗^*P* < 0.05 ^∗∗^*P* < 0.01 ^∗∗∗^*P* < 0.001).

The prerequisite for ensuring the effectiveness of anti-PD drugs is that they have excellent BBB permeability. Compared to other endothelial cell lines, bEnd.3 cells express higher expression of tight junction protein, which are more suitable to build an *in vitro* BBB model [27]. To test the BBB permeability of Fe-Cur NCPs, we implanted bEnd.3 cells into the insert part of Transwell to construct an *in vitro* BBB model. After the formation of a compact bEnd.3 monolayer membrane, we added free FITC and Fe-Cur-FITC to the upper chamber, and incubated for 2 h in dark, and then performed confocal imaging on both the bottom chamber and the insert chamber. First, the transepithelial electrical resistance (TEER) of Transwell before and after treatment with Fe-Cur-FITC and FITC remains stable, indicating that the tight bEnd.3 monolayer membrane in the Transwell was not damaged (Figure 3(b)). Interestingly, the fluorescence of the bottom chamber in the Fe-Cur-FITC group was significantly enhanced, showing about 3.9 times BBB permeability rate than the FITC group (Figures 3(a) and 3(c)). Moreover, as shown in Figures 3(d) and 3(e), significant time-dependent and concentration-dependent uptake of Fe-Cur-FITC by bEnd.3 cells was observed. Taken together, Fe-Cur NCPs can cross the BBB through noninvasive intracellular pathway. According to our previous study, the ultrasmall particle size of Fe-Cur NCPs is one of the reasons for the BBB crossing capability [28]. On the other hand, amphiphilicity of the nanomedicine endowed by PVP further enhances its brain delivery efficiency.

Endocytosis followed by transcytosis is the underlying mechanism for the transport of Fe-Cur NCPs across the BBB into the brain. We thus explored the uptake pathway of Fe-Cur-FITC by bEnd.3 cells. Firstly, bEnd.3 cells were incubated with Fe-Cur-FITC for 2 h at 37°C and 4°C. Since the production of intracellular ATP will be inhibited at a low temperature, the intracellular fluorescence intensities of the 37°C group were about twice that of the 4°C group, indicating an energy-dependent uptake process (Figures 3(f) and 3(g)). Furthermore, we added different uptake inhibitors including methyl-*β*-cyclodextrin (M*β*CD, caveolin inhibitor), chlorpromazine (CPZ, clathrin inhibitor), hypertonic sucrose (HS, clathrin inhibitor), or EIPA (macropinosome inhibitor) into the culture medium to investigate the penetration pathway of Fe-Cur-FITC through the bEnd.3 cell monolayer. Among them, high concentration of M*β*CD exhibited significant cytotoxicity (Figure S6), thereby we have chosen 2.5 mM for further experiment. As shown in Figures 3(h)–3(j), the above four inhibitors all showed significant inhibitory effects on the uptake of bEnd.3, indicating that Fe-Cur-FITC could be internalized by bEnd.3 cells through clathrin-mediated endocytosis, caveolin-mediated endocytosis, and macropinosome.

### 2.3. Examination of Cytotoxicity and *In Vitro* Neuroprotective Effect

**Figure 4:**
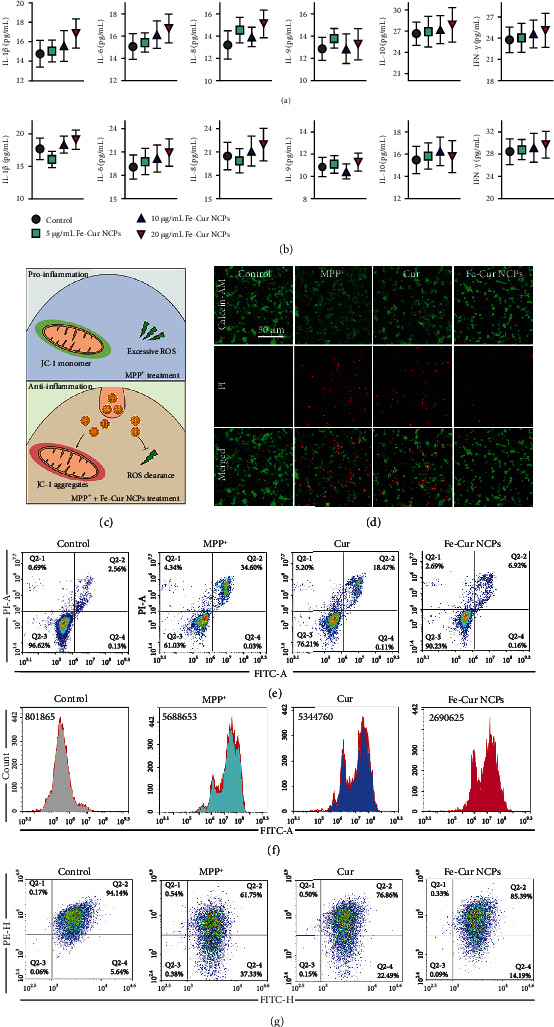
*In vitro* biocompatibility and neuroprotection evaluation. (a–b) Cytokine secretion from macrophages (a, SC cells; b, RAW264.7 cells) after treatment with different concentrations of Fe-Cur NCPs (*n* =3). (c) Schematic illustration of neuroprotective effects of Fe-Cur NCPs *in vitro*. (d) Live/dead staining images of SH-SY5Y cells after various treatments indicated. Scale bar: 20 *μ*m. (e–f) FACS analysis of Annexin V-FITC/PI-stained SH-SY5Y cells (e) and intracellular ROS (f) after various treatments. (g) Quantitative analysis of mitochondrial function using FACS, PE-H represents JC-1 aggregation, FITC-H represents JC-1 monomer.

There have been increasing evidences that nanomaterials can induce inflammatory responses by their conversion into protein corona complexes [29–31]. Therefore, we tested the proinflammatory effect of Fe-Cur NCPs on macrophages. Herein, we selected human-derived macrophages (SC cells) and macrophages obtained from leukemia virus transformed mouse cells (RAW264.7 cells) to conduct this study. Firstly, the cytotoxic effects of Fe-Cur NCPs were tested, with little cytotoxicity observed even at a high concentration of 80 *μ*M (in terms of Fe) (Figure S7). Next, we tested whether different concentrations of Fe-Cur NCPs (5, 10, and 20 *μ*M, in terms of Fe) could increase secretion of cytokines in SC and RAW264.7 cells. After cells were treated with NCPs, the culture supernatants were collected and analyzed using enzyme-linked immunosorbent assay (ELISA) kits for the detection of multiple cytokines (IL-1*β*, IL-6, IL-8, IL-9, IL-10, and IFN-*γ*). There was no significant difference in cytokine levels between the treatment groups and control group (no Fe-Cur NCPs treatment) (Figures 4(a) and 4(b)), indicating no proinflammatory effects of such therapeutic-based NCPs.

We further detected the intracellular uptake of NCPs via incubation of SH-SY5Y cells with ICG-labelled Fe-Cur NCPs. We found that the intracellular fluorescence intensities were increased with the increasing concentration and duration of incubation of Fe-Cur-ICG NCPs, showing both dosage and time-dependent properties (Figure S8). Next, we tested the cytotoxicity of Cur and Fe-Cur NCPs in SH-SY5Y cells and observed little cytotoxicity even at the concentration up to 20 *μ*M (Figure S9). Thus, Fe-Cur NCPs with concentrations of 5, 10, and 20 *μ*M were selected for further studying their neuroprotective effect. Specifically, we seeded SH-SY5Y cells expressing dopamine-*β*-hydroxylase and tyrosine hydroxylase into a 96-well plate, which were pretreated with a gradient concentration of Cur or Fe-Cur NCPs. Then, 1-Methyl-4-phenylpyridinium (MPP^+^), a neurotoxin by inhibiting mitochondrial redox functions, was added to build an *in vitro* PD model (Figure 4(c)). As shown in Figure 4(d), live/dead cell staining was performed to detect apoptosis in various groups. The MPP^+^ treated group showed the strongest red fluorescence of PI, while Cur could slightly relieve neurotoxicity induced by MPP^+^. Interestingly, after incubation of Fe-Cur NCPs, the cell viabilities of SH-SY5Y cells treated with MPP^+^ were increased to almost 100% without red fluorescence of PI, showing significant neuroprotective effect (Figure 4(d), Figure S10). Meanwhile, Annexin V-FITC/PI staining was further employed to evaluate the neuroprotective effect of Fe-Cur NCPs. Compared to free Cur, which showed slight neuroprotective effect, Fe-Cur NCPs treated group demonstrated the highest neuroprotective effect only with the apoptosis at 7.08%, consistent with the results of live/dead cell staining and CCK-8 assay (Figure 4(e)) [32]. Taken above results together, Fe-Cur NCPs showed much better neuroprotective effect than free Cur.

Since MPP^+^ could induce the oxidative stress and mitochondrial dysfunction of SH-SY5Y cells by increasing intracellular ROS level and reducing mitochondrial ATP production [33], we further investigated the mechanism of neuroprotective effect of Fe-Cur NCPs. Firstly, DCFH-DA, a probe showing green fluorescence in the presence of ROS, was employed to study the intracellular oxidative stress. As shown in Figure 4(f), SH-SY5Y cells after treatment with MPP^+^ inducing excessive intracellular ROS accumulation, free Cur even at the concentration of 20 M only slightly relieves the intracellular ROS level, whereas Fe-Cur NCPs could significantly decrease. In addition, the mitochondrial function of SH-SY5Y cells was also evaluated by the mitochondrial membrane potential (MMP) using JC-1 fluorescent probe (red, high MMP; green, low MMP) [34]. Unsurprisingly, cells treated with MPP^+^ exhibited considerable alterations in MMP, which could be significantly attenuated after Cur and Fe-Cur NCPs treatment. In particular, Fe-Cur NCPs could restore MMP level to normal in the MPP^+^ treated cells, indicating the significant protective effects on mitochondria (Figure 4(g), Figure S11). Therefore, we concluded that Fe-Cur NCPs may provide neuroprotection from the MPP^+^ treated cell by alleviation of oxidative stress and restoration of mitochondrial function.

### 2.4. Examination of Pharmacokinetics and Biodistribution

**Figure 5:**
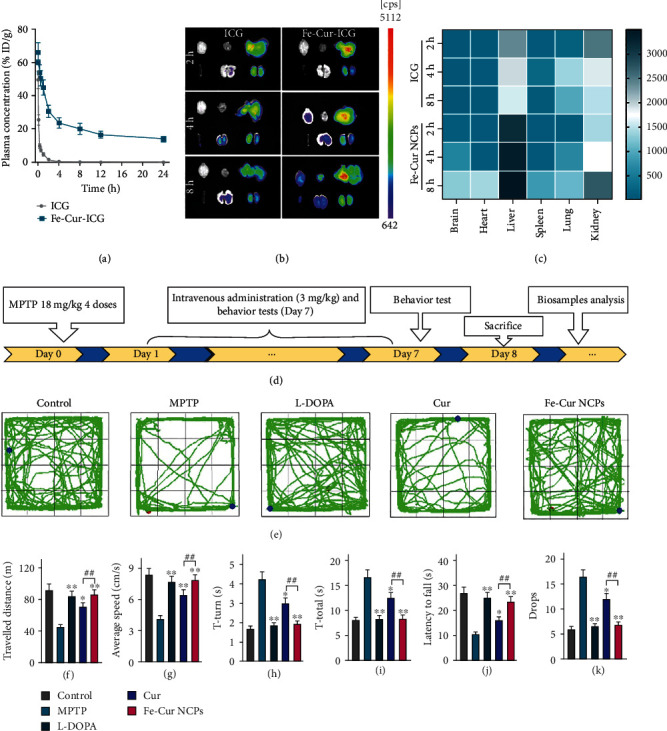
Biodistribution and *in vivo* anti-PD effect of Fe-Cur NCPs. (a) *In vivo* pharmacokinetic curves of free ICG and Fe-Cur-ICG NCPs after intravenous injection (*n* =4). (b) Fluorescence imaging of free ICG and Fe-Cur-ICG NCPs in the major organs at 2, 4, and 8 h after injection. (c) Fluorescence quantitative analysis of (b). (d) Schematic illustration of the pharmacodynamic study and behavior tests. For PD therapy, MPTP-induced PD mice were treated with 3 mg/kg of free Cur, 3 mg/kg of Fe-Cur NCPs and 25 mg/kg of L-DOPA (commonly prescribed medicine for Parkinson's). (e) Representative motive path of mouse activity (green). (f) Total distance travelled in the open-field test. (g) Average speed in the open-field test. (h–i) Time required for the mice to turn around (h) and descend (i) in the pole test. Latency time (j) and drop number (k) for falls from the rotating rod in the rotarod test (*n* = 8). Compared with the MPTP group: ^∗^*P* < 0.05 and ^∗∗^*P* < 0.01. Compared with the Cur group: ^#^*P* < 0.05 and ^##^*P* < 0.01.

A long half-life is necessary for ensuring good therapeutic efficacy. We hypothesized that ultrasmall NCPs have a longer half-life than free therapeutics. Thus, we measured the pharmacokinetics of Fe-Cur-ICG NCPs and free ICG in the blood. As shown in Figure 5(a), Fe-Cur-ICG NCPs exhibited significantly improved systemic circulation in contrast to free ICG, remaining at a relatively high concentration (14.09% ID/g) at 24 h post-injection (p.i.). The long circulation of Fe-Cur-ICG NCPs is derived from PVP, which reduces the combination of nanomedicine and protein during systemic circulation [35], thus weaken the formation of nano-corona complex and thereby easing the clearance of immune cells. It was worth mentioning that the blood circulation of Fe-Cur-ICG NCPs showed a two-compartment model with the first and second phases of blood clearance of 0.86 ± 0.12 h and 18.16 ± 1.95 h, respectively.

Besides, the accumulations of drugs in the disease site also affect drug efficacy, especially in brain diseases wherein drugs need to cross the BBB. After intravenously (i.v.) injection of Fe-Cur-ICG NCPs into mice, the major organs including the brain, heart, liver, spleen, lung, and kidney were collected at 2, 4, and 8 h p.i. and imaged by *in vivo* fluorescence imaging. Fe-Cur-ICG NCPs showed significant fluorescence signals in the brain at 4 h and 8 h, which was 22.9 and 74 times than that of free ICG at the respective time points (Figures 5(b) and 5(c)), demonstrating the excellent capacity to cross the blood-brain barrier (BBB). Interestingly, obvious fluorescence signals in the kidneys were observed by in vivo imaging at 2, 4, and 8 h post-injection of Fe-Cur-ICG NCPs, indicating that such Fe-Cur-ICG NCPs could be cleared out from the mouse body via renal pathway, which is favorable to biosafety and the future clinical translation.

### 2.5. Examination of *In Vivo* Effect against MPTP-Induced Parkinsonism

MPTP approach was selected to build the animal model of PD (Figure 5(d), Figure S12). To assess the pharmacodynamics of Fe-Cur NCPs, we examined treated mice using a variety of behavioral assays, including the pole test, rotarod test, and open-field test. In the open-field test, the mice were moved freely in an open field, and the movement trajectory and movement speed were recorded to evaluate the general motor function of the mice. The travelled distance and average speed of the mice were the lowest in the MPTP group. Notably, after i.v. injection of Fe-Cur NCPs, MPTP-treated mice showed the greatest improvement in exercise ability (Figures 5(e)–5(g)), similar to the healthy mice (control group). It was worth mentioning that only 3 mg/kg dose of Fe-Cur NCPs showed highly effective treatment, comparable to that of a high dose of L-DOPA (25 mg/kg). Furthermore, the pole test is used to measure the motor skill and MPTP-induced bradykinesia. Mice treated with MPTP showed a huge increase in the T-turn and T-total compared to the healthy control mice. Interestingly, compared to free Cur, Fe-Cur NCPs could significantly reduce the T-turn and T-total, even gradually return to normal levels in the pole test (Figures 5(h) and 5(i)).

To test the balance and coordination, we also recorded the fall latency and the total number of drops in the rotarod test. We observed that MPTP significantly reduced the latency time for falls and increased the number of drops. While free Cur was only able to slightly increase the latency time and decrease the number of drops in the PD mice, Fe-Cur NCPs could restore the balance and coordination ability in mouse models of PD to the level observed in healthy mice (Figures 5(j) and 5(k)). The above data showed that Fe-Cur NCPs could be effective in relieving motor dysfunction observed in PD.

### 2.6. Examination of the Neuroprotective Mechanism of Fe-Cur NCPs

**Figure 6:**
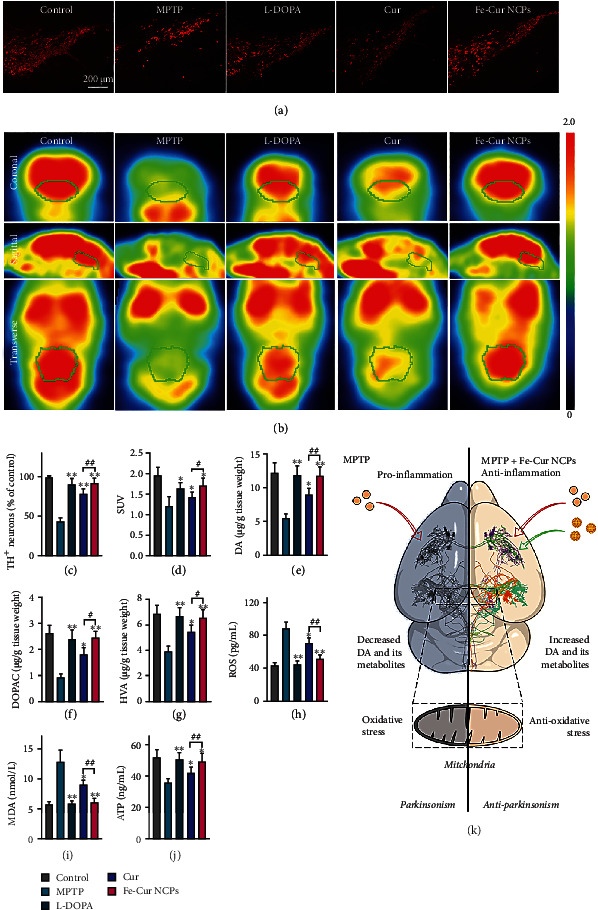
The neuroprotective mechanism of Fe-Cur NCPs. (a) Representative images of sections stained for TH^+^ neurons using immunohistochemistry after various treatments. Scale bar: 200 *μ*m. (b) [18F]-FDG PET imaging of the brain after various treatments (brain stem inside the white circle). (c) Levels of DA Quantification of TH^+^ neurons in the SNpc (*n* =3). (d) [18F]-FDG uptake in the left striatum (*n* =3). Compared with the MPTP group: ^∗^*P* < 0.05 and ^∗∗^*P* < 0.01. (e–g) The levels of DA, DOPAC, and HVA in the striatum (*n* =3). (h–j) The levels of ROS, MDA, and ATP in the midbrain after various treatments were indicated (*n* =3). (k) Schematic illustration of anti-Parkinsonism mechanism. In the above experiments, MPTP-induced PD mice were treated with 3 mg/kg of free Cur, 3 mg/kg of Fe-Cur NCPs, and 25 mg/kg of L-DOPA (commonly prescribed medicine for PD).

MPTP has been shown to induce PD-like symptoms such as striatal oxidative stress, elevated malondialdehyde (MDA) levels, and TH^+^ neuron death [36]. To further study the mechanism underlying the effects of Fe-Cur NCPs against PD dyskinesia, we dissected out the midbrain and striatum of treated mice under anesthesia. Next, the sections of the isolated brain tissues were stained by immunofluorescence assays to estimate the number of TH^+^ neurons (dopaminergic neurons) in the substantia nigra pars compacta (SNpc). The total amount of red fluorescence was the lowest in the MPTP-treated group, indicating a huge loss of TH^+^ neurons compared to healthy mice (Figure 6(a), Figure S13). Free Cur could moderately increase the number of TH^+^ neurons, whereas after treatment of 3 mg/kg of Fe-Cur NCPs, the number of TH^+^ neurons was returned to the normal level, comparable to that of 25 mg/kg L-DOPA. Cell counts revealed that the number of TH^+^ neurons in the SNpc after MPTP treatment was only 42.93% of that in the control group, while in the L-DOPA (25 mg/kg), free Cur (3 mg/kg), and Fe-Cur NCPs groups (3 mg/kg), this value was 90.38%, 78.06%, and 91.55%, respectively (Figure 6(c)).

Furthermore, the [^18^F]-FDG, an indicator of brain glucose metabolism that is commonly used in clinical settings to track the development of PD [37], was used as a contrast agent for positron emission tomography (PET) imaging in each group of mice [38]. The signal intensity in the brain stem of the MPTP group was significantly reduced, indicating that MPTP inhibited glucose metabolism in the brain. After treatment of free Cur, L-DOPA, and Fe-Cur NCPs, the energy metabolism in the brain of PD mice was improved observed by [^18^F]-FDG PET imaging, where Fe-Cur NCPs showed the best significant therapeutic effect (Figure 6(b)). Through a quantitative analysis of the [^18^F]-FDG signal in the brainstem, we further found that the standardized uptake values (SUV) in the control, MPTP, L-DOPA, free Cur, and Fe-Cur NCPs groups were 1.96, 1.21, 1.63, 1.42, and 1.71, respectively (Figure 6(d)). These results indicated that Fe-Cur NCPs could return the loss of TH^+^ neurons and provide an excellent neuroprotective effect.

Since MPTP-induced neurotoxicity is associated with aberrant DA metabolism, we examined the levels of DA and its metabolites homovanillic acid (HVA) and 3,4-dihydroxyphenylacetic acid in the collected striatum tissues (DOPAC) [39]. The levels of DA, DOPAC, and HVA in the striatum from MPTP-treated PD mice dropped to 44.6%, 35.9%, and 57.5% compared to healthy control mice. After treatment 3 mg/kg Fe-Cur NCPs or 25 mg/kg commercial L-DOPA, the levels of DA, DOPAC, and HVA in the striatum of PD mice were recovered to normal level (Figures 6(e)–6(g)), indicating that Fe-Cur NCPs could significantly attenuate the damage to DA metabolism.

In addition, we set out to confirm that Fe-Cur NCPs can also effectively reduce excessive ROS levels, relieve oxidative stress, and improve mitochondrial function *in vivo*, through quantitative analysis of ROS, lipid metabolite MDA, and ATP in the midbrain. As shown in Figures 6(h)–6(j), the levels of ROS and MDA in the midbrain from MPTP-treated PD mice were increased significantly to 203.7% and 224.6% in comparison to healthy mice, resulting in mitochondrial dysfunction and reduced ATP production. Among all treatment groups, Fe-Cur NCPs group showed the highest antioxidant and neuroprotection effects, in which the ROS and MDA levels restored to 119% and 106.1% of control levels, respectively, while the ATP levels returned to 94.9%. The outstanding anti-Parkinsonism effect of Fe-Cur NCPs could be attributed to not only prolonged blood circulation, enhanced crossing BBB, and improved therapeutic accumulation in the brain but also significant antioxidant and neuroprotection effect (Figure 6(k)).

Collectively, Fe-Cur NCPs showed a certain extent of recovery in the dopaminergic system and behaviors. Fe-Cur NCPs also ameliorated energy metabolism in the brain of PD mice via scavenging excess ROS, which realized neuroprotection against PD.

### 2.7. Assessment of Biocompatibility

*In vivo* biosafety profiles of nanomedicine are critical for future clinical translations. Even though our *in vitro* experiments repeatedly demonstrated the biosafety of Fe-Cur NCPs, the *in vivo* examination was further investigated. Specifically, we conducted a 4-week investigation on the safety of Fe-Cur NCPs utilizing hematological tests and hematoxylin and eosin (H&E) staining to better elucidate the in vivo biosafety. Firstly, we intravenously injected a high dose of Fe-Cur NCPs (10 mg/kg) into mice, then the blood sample and major organs at days 1, 7, and 28 were collected for blood biochemistry and complete blood panel analysis and H&E staining, respectively. As shown in Figure S14A-C, there was no variation in the hematological index and blood biochemistry (e.g., alanine aminotransferase, ALT; aspartate aminotransferase, AST; Tot Prot, TP; globulin, GLB; blood urea nitrogen, BUN; creatinine, CREA; red blood cell, RBC; white blood cell, WBC; platelet, PLT; neutrophil, Neu; hemoglobin, HGB; hematocrit, HCT) between healthy and Fe-Cur NCP-treated animals on days 1, 7, or 28. Furthermore, major organs including the heart, liver, spleen, lung, and kidney from mice treated with Fe-Cur NCPs also showed no obvious tissue damages and adverse effects (Figure S14D). Taken together, our findings indicate that Fe-Cur NCPs are extremely safe in vivo and may be employed for further in vivo PD therapy.

Furthermore, a more comprehensive evaluation of the *in vivo* biocompatibility of Fe-Cur NCPs in the MPTP-induced PD mice was also tested. During the treatment period, we found that the weights of MPTP-induced PD mice were decreased, whereas Fe-Cur NCPs could partly increase bodyweights of PD mice (Figure S15). H&E staining of the major organs (e.g., the heart, liver, spleen, lung, and kidney) showed that Fe-Cur NCPs did not cause obvious tissue damages or adverse effects in these organs (Figure S16). Furthermore, regular blood measurements and biochemical indicators of liver and kidney function also demonstrated that Fe-Cur NCPs did not produce systemic damage to the MPTP-induced PD mice (Figure S17).

In addition, we also examined whether the accumulation of Fe-Cur NCPs in the brain after treatment induced any significant additional effects, such as cerebral thrombosis. Thus, we performed nuclear magnetic resonance imaging (MRI) of the mice brains to obtain T2-weighted images (Figure S18), demonstrating that Fe-Cur NCPs have no significant side effect on the brain. In summary, Fe-Cur NCPs showed excellent biocompatibility, appropriate for the treatment of PD.

## 3. Conclusions

Relieving the neuroinflammation through anti-inflammatory reagents is an emerging strategy to treat PD. However, due to the limited BBB permeability and poor half-life, most of the reagents cannot exhibit significant curative effects *in vivo*. Herein, we propose a curcumin-based NCP for effective PD therapy by prolonging blood circulation and enhancing BBB crossing, as well as removing excessive ROS and relieving inflammatory condition in PD brains. Both *in vitro* and *in vivo* experiments demonstrated that Fe-Cur NCPs could alleviate the MPP^+^/MPTP-induced PD symptoms by removing excessive ROS and improving mitochondrial functions to achieve neuroprotection. Meanwhile, by using immunofluorescence and PET to examine the effects of these NCPs on the number of TH^+^ neurons and glucose metabolism in the brain, respectively, we confirmed that Fe-Cur NCPs could inhibit the MPTP-induced loss of TH^+^ neurons. These curative effects of Fe-Cur NCPs were attributed to significant anti-inflammatory properties of the curcumin and multiple enzymes (e.g., catalase, superoxide dismutase)-like activities of the Fe-based nanozymes. Furthermore, through multiple safety evaluation experiments, including the cytotoxicity assays, analysis of proinflammatory effects, H&E staining of the major organs, brain MRI, and assessment of blood routine and biochemical indicators, we have demonstrated that Fe-Cur NCPs have excellent biocompatibility. Therefore, Fe-Cur NCPs hold promise as a novel, safe, and efficient advanced therapeutic agent for PD.

## 4. Materials and Methods

### 4.1. Materials

FeCl_3_•6H_2_O 2,2-diphenyl-1-picrylhydrazyl (DPPH), 2'-Azinobis-(3-ethylbenzthiazoline-6-sulphonate) (ABTS), and MB were procured from J&K Scientific Co., Ltd (Beijing, China). Polyvinylpyrrolidone (PVP, MW =8000) was purchased from Titan Scientific Co., Ltd (Shanghai, China). Cell Counting Kit-8 (CCK-8) was obtained from Dojindo Laboratories (Kumamoto, Japan). The Calcein-AM/PI double staining kit and 2,7-dichlorodi-hydrofluorescein diacetate (DCFH-DA) were procured from Beyotime Biotechnology Co., Ltd. (Beijing, China). 1-methyl-4-phenyl-1,2,3,6-tetrahydropyridine (MPTP) was obtained from MedChemExpress (NJ, USA). Cur, Indocyanine Green (ICG), 1-methyl-4-phenylpyridinium ion (MPP^+^), and Levodopa (L-DOPA) were obtained from Sigma-Aldrich (St. Louis, USA). Reactive oxygen species (ROS), adenosine triphosphate (ATP), and malondialdehyde (MDA) ELISA kits were purchased from R&D Systems, Inc., USA.

### 4.2. Cells and Animal Models

Human macrophages obtained from peripheral blood (SC), macrophages obtained from leukemia virus transformed mouse cells (RAW264.7), and human neuroblastoma cells (SH-SY5Y) were cultured in DMEM containing 1% streptomycin/penicillin together with 10% FBS in a 37°C humidified 5% CO_2_ incubator.

For animal studies, the Experimental Animal Center of Guangzhou University of Chinese Medicine provided male 7-week-old Sprague-Dawley (SD) rats (180-200 g) and 8-week-old C57BL/6 mice (22 - 26 g), housed with *ad libitum* food/water under specific pathogen-free conditions. The Animal Ethics Committee of Guangzhou University of Chinese Medicine approved such studies, designed as per government-approved animal care and use guidelines.

### 4.3. Synthesis of Fe-Cur NCPs

First, 1 mL of FeCl_3_•6H_2_O methanol solution (20 mg/mL) was added dropwise to 5 mL of PVP methanol solution (13.2 mg/mL). After continuous stirring for 5 min, 10 mg Cur in 1 mL methanol was added dropwise, and the mixture was incubated at room temperature for 3 h under stirring. The resulting methanol solution was dialyzed overnight with water, and the Fe-Cur NCPs obtained were collected. To prepare the Fe-Cur-ICG or Fe-Cur-FITC, 1 mg ICG or 100 *μ*g FITC was added with 10 mg Cur in 1 mL methanol followed by the same operation.

### 4.4. Characterization of Fe-Cur NCPs

A DLS approach was used to assess the hydrodynamic diameter and zeta potential of the Fe-Cur NCPs with a ZetaSizer Nano ZS instrument (Malvern, UK). A TEM instrument (FEI Talos F200X) was used to characterize the shape and structure of these NCPs, while a HI Quantera SXM instrument equipped with an Al X-ray excitation source was utilized for X-ray photoelectron spectroscopy (XPS) measurements. A SpectraMAX M2 microplate reader (Molecular Devices) was used to assess the Vis-NIR absorbance spectrum of Fe-Cur NCPs, while inductively coupled plasma atomic emission spectrometry (ICP-OES) was implemented to evaluate iron levels within these NCPs.

### 4.5. ROS Scavenging Assays

DPPH, ABTS, and MB assays were employed for evaluating the ROS scavenging ability of these NCPs. In the DPPH assay, a solution of DPPH in ethanol (final concentration: 62.5 M) was combined with a range of Fe-Cur NCPs concentrations (0, 1.5625, 3.125, 6.25, 12.5, and 25 *μ*g/mL). After mixing completely, this mixture was placed into incubation (30 min and room temperature), following which absorption at 510 nm was measured, and DPPH scavenging activity was assessed as follows: DPPH scavenging ability (%) = (A_DPPH_-A_sample_)/A_DPPH_) ∗ 100%, where A_DPPH_ and A_Sample_ represent the DPPH absorbance in the absence of additional treatment and after the addition of Fe-Cur NCPs, respectively, [40].

For ABTS assays, ABTS solution (7 mM) was incubated with potassium persulfate (2.45 mM) overnight to activate ABTS free radicals. This radical solution was then combined with a dose range of Fe-Cur NCPs (0, 1.5625, 3.125, 6.25, 12.5, and 25 *μ*g/mL). After a 10 min incubation, ABTS absorption was measured at 510 nm, with scavenging ability then being quantified as follows: ABTS scavenging ability (%) = (A_ABTS_-A_sample_)/A_ABTS_) ∗ 100%, where A_ABTS_ and A_Sample_, respectively, correspond to the ABTS absorbance for untreated samples and for samples to which Fe-Cur NCPs had been added [41].

Fenton reaction-derived •OH radicals can bleach MB, and as such, MB can be analyzed to gauge the ability of Fe-Cur NCPs to scavenge these hydroxyl radicals. As such, MB was combined with a dose range of Fe-Cur NCPs (3.125, 6.25, 12.5, 25, and 50 *μ*g/mL). This mixture was then combined with a Fenton reaction solution containing H_2_O_2_ and Fe^2+^. Following incubation for 15 min at room temperature, MB absorption was measured, and MB scavenging ability was quantified as follows: MB scavenging ability (%) = (A_sample_/A_MB_) ∗ 100%, where A_MB_ and A_sample_, respectively, correspond to MB absorbance without further treatment and following Fe-Cur NCPs addition [42].

### 4.6. Cytotoxicity and Cytokine Secretion Analyses

Following incubation for 24 h in 96-well plates (1.0 × 10^5^ cells per well), SC and RAW264.7 cells were treated with a range of Fe-Cur NCPs concentrations (Cur concentrations of 5, 10, 20, 40, and 80 *μ*M) for an additional 24 h period. Subsequently, cytotoxicity and cytokine levels were detected using CCK-8 and ELISA kits, respectively [43].

### 4.7. *In Vitro* BBB Model and Uptake Mechanism

After b.End.3 cells plating in 24-well Transwell chambers at 5 × 10^4^ cells/well, media in the upper chamber was renewed every day while media in the lower chamber was changed every other day. The *in vitro* BBB model was successfully established one week later. For BBB permeability analysis, the cells were incubated with free FITC or Fe-Cur-FITC for 2 h. Next, the FITC concentration was detected by UV-Vis spectrometer, and the permeability ratio was calculated via the following formula:

(1) $$\begin{array}{c} P\left(\%\right)=\left(1-\frac{C_{1}}{C_{0}}\;\right)\times 100\%, \end{array}$$

where *P* (%) refers to the permeability ratio, *C*_0_ means the initial concentration of FITC at the top chamber, *C*_1_ is the concentration of FITC at the top chamber after incubation.

For time-dependent uptake experiments, bEnd.3 cells were added to 12-well plates (7 × 10^4^ cells/well). DMEM containing 10 *μ*M Fe-Cur-FITC was then added to these wells, followed by incubation for 1, 2, or 4 h. Cultures were subsequently washed with PBS, fixed with 4% paraformaldehyde, stained using DAPI, and finally imaged confocal microscope. For concentration-dependent assays, after plating as above, cells were treated using DMEM supplemented with a range of Fe-Cur-FITC concentrations (5, 10, and 20 *μ*M) for 2 h, followed by washing, fixation, DAPI staining, and confocal imaging as above. For uptake mechanism-focused analyses, cells were preprocessed with 2.5 mM M*β*CD, 30 mM CPZ, 0.4 M HS, and 40 *μ*M EIPA for 30 min, with subsequent 10 *μ*M Fe-Cur-FITC exposure for 2 h. Cells were then rinsed, fixed, DAPI stained, and imaged as above.

### 4.8. Cellular Uptake of Fe-Cur-ICG NCPs

ICG-labelled NCPs (Fe-Cur-ICG NCPs) were used for the visualization of endocytic uptake of NCPs. Initially, SH-SY5Y cells were seeded overnight within 12-well plates (1 × 10^5^ cells/well), followed by subsequent triple PBS-rinse and eventual incubation at 37°C in serum-free media containing Fe-Cur-ICG NCPs at final ICG concentrations of 10 *μ*g/mL, 20 *μ*g/mL, or 40 *μ*g/mL. Following an appropriate incubation period, cells were harvested, rinsed with chilled PBS, fixed with 4% paraformaldehyde, and visualized using confocal laser scanning microscope (CLSM; TCS SPE II^@^, Leica™, Germany).

### 4.9. *In Vitro* Analyses of Neuroprotection Effects

#### 4.9.1. Cell Viability

The cytotoxic effects of Fe-Cur NCPs against SH-SY5Y cells were assessed by seeding these cells within 96-well plate (5 × 10^3^ cells/well) for one day. Cultures were then treated for an additional 24 h with a range of Fe-Cur NCPs concentrations (Cur concentrations of 0, 5, 10, 20, 40, 80, or 160 *μ*M). A CCK-8 assay was then used to quantify cytotoxicity.

#### 4.9.2. Flow Cytometry Analyses

The neuroprotective effect of Fe-Cur NCPs was assessed by plating SH-SY5Y cells within 96-well plates (5 × 10^3^ cells/well). Following a 24 h incubation, these cells further exposed (2 h) with a range of Cur or Fe-Cur NCPs concentrations (Cur concentrations: 5, 10, and 20 *μ*M). Then, cells were treated with 2 mM MPP^+^ incubated in this condition for 36 h. Cultures were then collected, resuspended, and stained using Annexin V-FITC (5 *μ*L) and propidium iodide (PI, 10 *μ*L), and apoptotic death was assessed via FACS [44].

#### 4.9.3. Intracellular ROS Detection

Followed pretreatment with Cur or Fe-Cur NCPs (20 *μ*M) for 2 h, SH-SY5Y cells were treated with 2 mM MPP^+^ and incubated for 36 h. DCFH-DA was then added as a fluorescent probe for ROS detection [36], and the cells were quantified via flow cytometry.

#### 4.9.4. MMP Detection

Following reoxygenation, cells were harvested and resuspended in PBS. According to the manufacturer's protocol, a working solution of JC-1 staining was then prepared and added to cells for 15 min at 37°C. Cells were then rinsed three times using a working buffer, followed by imaging with a flow cytometer [45].

### 4.10. Pharmacokinetics and Biodistribution

Nanocarrier half-lives in systemic circulation were assessed by intravenously injecting mice (*n* =3) with either free ICG or Fe-Cur-ICG NCPs (100 *μ*L). At 0.083, 0.167, 0.333, 0.5, 1, 2, 4, 6, 8, 12, and 24 h post-injection, retro-orbital blood samples were collected from these mice, and ICG concentrations in these samples were measured with a UV-vis spectrometer based on absorbance spectra for solubilized blood samples. The ICG equivalent for Fe-Cur-ICG NCPs was established as a percentage of administered dose/gram of tissue (% ID/g). An independent group of animals subjected to identical treatment conditions were used for ex vivo fluorescent imaging of major organs (heart, lungs, kidneys, spleen, liver). For these imaging biodistribution analyses, animals were anesthetized and perfused with 0.9% saline at 2, 4, or 8 h post-injection, after which these organs were harvested and imaged.

### 4.11. *In Vivo* Analyses of Neuroprotection Effects

#### 4.11.1. Establishment of PD Model and Treatment

Male C57BL/6 mice were randomly allocated to the control, MPTP, L-DOPA, Cur, and Fe-Cur NCPs treatment groups. To establish the PD model, MPTP (18 mg/kg) solution was intraperitoneally injected four times (every 2 h) [46], while control mice were administered an equivalent volume of 0.9% saline. During the following week, mice were treated once every two days with 0.9% saline (i.v., control and MPTP groups), L-DOPA (i.p., 25 mg/kg, L-DOPA group), Cur (i.v., 3 mg/kg, Cur group), or Fe-Cur NCPs (i.v., Cur dose of 3 mg/kg, Fe-Cur NCP group).

#### 4.11.2. Behavioral Tests

Following treatment, a series of pole, rotarod, and open-field tests were used to analyze murine behavior to gauge neuroprotective efficacy [47]. For pole studies, mice were placed on a vertical pole (d: 1 cm, h: 50 cm) that had a rugged terrain, with turn-head time (T-turn) being recorded, as was the time needed by these animals to reach the bottom of the pole (T-total). For rotarod testing, animals were placed on individual rotarod sections (20 rpm/120 s), with falling time and the overall fall events being recorded. For open-field testing, murines were positioned centrally within a 60 cm x 60 cm x 40 cm monitoring chamber, and their movement over a 20 min period was recorded, with speed determined quantitatively through open-field software (Clever Sys Inc.™, USA).

#### 4.11.3. Immunofluorescence

After mice had been euthanized, midbrain sections were harvested for subsequent analyses. TH neurons in SNpc were quantified by preparing sequential coronal sections (30 *μ*m/AP -2.80 to -3.97 mm). These sections were then stained with primary rabbit anti-TH (Abcam™, 1 : 1000) followed by secondary Alexa Fluor 594-conjugated anti-rabbit IgG (Cell signaling Technology™, 1 : 1000). Cells were then imaged with a fluorescent microscope (Model DMi8®, Leica™, Germany), and ImageJ was employed for quantifying TH neuron numbers [48].

#### 4.11.4. PET/CT Imaging

Energy metabolism in the brains of these mice was assessed via a PET/CT imaging approach on day 9 of the treatment period. Murines were placed into fasting for 12 h prior to PET imaging, followed by the i.v. administration of [^18^F]-FDG (200 ± 10 *μ*Ci). After 1 h, these mice were then anesthetized using isoflurane (2%) and transferred to the scanning bed, with imaging being performed in static mode (10 min). CT scans within normal mode were performed through TransPET Discoverist 180® platform (Raycan Technology™, China). The 3D-OSEM methodology was utilized for reconstructing PET images (voxel size: 0.5 × 0.5 × 0.5 mm^3^). CT image reconstruction was performed through FDK algorithm (256 × 256 × 256 matrices). Images were visualized with the AMIDE and Pmod (Pmod Technologies LLC™, Switzerland) software, with the mean standardized uptake value (SUV) being computed based on the equation: mean pixel value for the decay-corrected region-of-interest activity (*μ*Ci/kg)/(injected dose [*μ*Ci]/weight [kg]) [32].

#### 4.11.5. Detection of Striatal DA Levels

Striatum samples were harvested and prepared as previously described [49]. DA levels together with investigated metabolites 3,4-dihydroxyphenylacetic acid (DOPAC) and homovanillic acid (HVA) were detected using an ESA chromatograph with a 5014B electrochemical detector.

#### 4.11.6. Midbrain Mitochondrial Function Analyses

Midbrain mitochondrial function was evaluated using an established method [50]. Briefly, midbrain samples were immediately harvested, homogenized within ice-cold 0.9% saline, and placed for centrifuging for 10 min (4°C/1000 xg). Supernatants were consequently harvested for ELISA-based analyses of ROS, ATP, and MDA levels [51].

### 4.12. Biocompatibility Evaluation

As discussed above, healthy mice received four intravenous injections of Fe-Cur NCPs using Cur dose of 3 mg/Kg. Murine brain imaging was then conducted via nuclear magnetic resonance (NMR; 9.4 T Biospec MRI®, Bruker™, Germany). Blood samples were additionally harvested from these animals for biochemical analyses, and major organs (heart, lungs, kidneys, spleen, liver) were collected for H&E staining.

Prior to *in vivo* pharmacodynamic and pharmacokinetic experiments, Fe-Cur NCP *in vivo* toxicity was assessed by randomly assigning mice to control of Fe-Cur NCP treatment groups. Mice in the Fe-Cur NCP group were administered these NCPs (i.v.) at a 10 mg/kg Cur dose, while control animals were administered an equivalent volume of 0.9% saline. At 1, 7, and 28 days post-injection, major organs and blood were collected from these animals as above for histological and biochemical analyses, respectively. Histological analyses were performed by fixing organs (heart, lungs, kidneys, spleen, liver) using 4% paraformaldehyde followed by paraffin embedding. These samples were then cut to yield 4 *μ*m sections, and were subjected to H&E staining.

### 4.13. Statistical Analysis

All data are means ± standard deviations (SDs) from independent assessments, and were studied via Student's *t*-tests or one-way ANOVAs with the Newman-Keuls post hoc assessment, as appropriate. *P* < 0.05 was the threshold of significance for this study.

## Data Availability

All data used to support the findings in the paper and supplementary materials are available from the corresponding authors upon reasonable request.

## Supplementary Materials

Supplementary Materials Figure S1. Colloid stability of Fe-Cur NCPs (a) in fetal bovine serum (FBS) at 37°C for 24 h and (b) in PBS at 4°C for 7 days. Figure S2. X-ray diffraction (XRD) spectrum of Fe-Cur NCPs. Figure S3. X-ray photoelectron spectroscopy (XPS) spectra of Fe-Cur NCPs. Figure S4. (a) Particle size, (b) PDI, and (c) zeta potential of Fe-Cur NCPs after labelled by FITC (Fe-Cur-FITC NCPs) or ICG (Fe-Cur-ICG NCPs). Figure S5. Absorbance spectra of methylene blue (MB) after treatment with different concentrations of Fe-Cur NCPs. Figure S6. Cytotoxicity after treatment with different concentrations of M*β*CD to bEnd.3 cells. Figure S7. Cytotoxicity analysis in RAW264.7 (A) and SC (B) cells treated with different concentrations of Fe-Cur NCPs (*n* =3). Figure S8. Cellular uptake of Fe-Cur-ICG NCPs in SH-SY5Y cells. (A) Concentration-dependent images after incubation for 1 h. (B) Time-dependent images after incubation with 20 *μ*g/mL Fe-Cur-ICG NCPs. Figure S9. Cytotoxicity analysis in SH-SY5Y cells treated with different concentrations of Cur or Fe-Cur NCPs (*n* =3). Figure S10. Cell viability of SH-SY5Y cells treated with MPP^+^ and different concentrations of Cur or Fe-Cur NCPs (*n* =3). Figure S11. The effects of Cur and Fe-Cur NCPs (both Cur concentration of 20 *μ*M) on mitochondrial membrane potential (*n* =3). Figure S12. Schematic illustration of pharmacodynamic study and performances. Figure S13. Anatomical location of the substantia nigra pars compacta (SNpc). Figure S14. *In vivo* toxicity evaluation. (A-C) Hematological indexes and blood biochemistry (alanine aminotransferase, ALT; aspartate aminotransferase, AST; total protein, TP; globulin, GLB; blood urea nitrogen, BUN; creatinine, CREA; red blood cell, RBC; white blood cell, WBC; platelet, PLT; neutrophil, Neu; hemoglobin, HGB; hematocrit, HCT) examined in mice treated with Fe-Cur NCPs (10 mg/kg) at (A) day 1, (B) day 7, and (C) day 28 (*n* =3). (D) Histological examinations of the main organs (heart, liver, spleen, lung, and kidney) of mice treated with Fe-Cur NCPs (10 mg/kg) at different time points using H&E staining. Figure S15. Body weights of PD mice during various therapeutic treatments indicated. Figure S16. H&E-stained images of the indicated organs of PD mice in different treatment groups. Figure S17. Hematological parameters and blood biochemical analyses of the PD mice treated with different groups (*n* =6). Figure S18. MRI scans of mice brains in control and Fe-Cur NCPs groups. Scale bar: 200 *μ*m.
