# Supplementary material for: Ultrasmall Coordination Polymers for Alleviating ROS-Mediated Inflammatory and Realizing Neuroprotection against Parkinson's Disease
Source: Research (Wash D C). 2022 Jul 18;2022:9781323. doi: 10.34133/2022/9781323 (PMC9343083; doi:10.34133/2022/9781323)
Supplement: Supplementary Materials — Figure S1. Colloid stability of Fe-Cur NCPs (a) in fetal bovine serum (FBS) at 37°C for 24 h and (b) in PBS at 4°C for 7 days. Figure S2. X-ray diffraction (XRD) spectrum of Fe-Cur NCPs. Figure S3. X-ray photoelectron spectroscopy (XPS) spectra of Fe-Cur NCPs. Figure S4. (a) Particle size, (b) PDI, and (c) zeta potential of Fe-Cur NCPs after labelled by FITC (Fe-Cur-FITC NCPs) or ICG (Fe-Cur-ICG NCPs). Figure S5. Absorbance spectra of methylene blue (MB) after treatment with different concentrations of Fe-Cur NCPs. Figure S6. Cytotoxicity after treatment with different concentrations of MβCD to bEnd.3 cells. Figure S7. Cytotoxicity analysis in RAW264.7 (A) and SC (B) cells treated with different concentrations of Fe-Cur NCPs (n =3). Figure S8. Cellular uptake of Fe-Cur-ICG NCPs in SH-SY5Y cells. (A) Concentration-dependent images after incubation for 1 h. (B) Time-dependent images after incubation with 20 μg/mL Fe-Cur-ICG NCPs. Figure S9. Cytotoxicity analysis in SH-SY5Y cells treated with different concentrations of Cur or Fe-Cur NCPs (n =3). Figure S10. Cell viability of SH-SY5Y cells treated with MPP+ and different concentrations of Cur or Fe-Cur NCPs (n =3). Figure S11. The effects of Cur and Fe-Cur NCPs (both Cur concentration of 20 μM) on mitochondrial membrane potential (n =3). Figure S12. Schematic illustration of pharmacodynamic study and performances. Figure S13. Anatomical location of the substantia nigra pars compacta (SNpc). Figure S14. In vivo toxicity evaluation. (A-C) Hematological indexes and blood biochemistry (alanine aminotransferase, ALT; aspartate aminotransferase, AST; total protein, TP; globulin, GLB; blood urea nitrogen, BUN; creatinine, CREA; red blood cell, RBC; white blood cell, WBC; platelet, PLT; neutrophil, Neu; hemoglobin, HGB; hematocrit, HCT) examined in mice treated with Fe-Cur NCPs (10 mg/kg) at (A) day 1, (B) day 7, and (C) day 28 (n =3). (D) Histological examinations of the main organs (heart, liver, spleen, lung, and kidney) of [file 9781323.f1.docx]

Supplementary Materials

**Ultrasmall Coordination Polymers for Alleviating ROS-Mediated Inflammatory and Realizing Neuroprotection against Parkinson’s Disease**

Guowang Cheng^1#^, Xueliang Liu^3#^, Yujing Liu^2^, Yao Liu^2^, Rui Ma^2^, Jingshan Luo^2^, Xinyi Zhou^3,5^, Zhenfeng Wu^1*^, Zhuang Liu^4^, Tongkai Chen^2*^, Yu Yang^3*^

**This file include:**

Figures S1 to S18

**
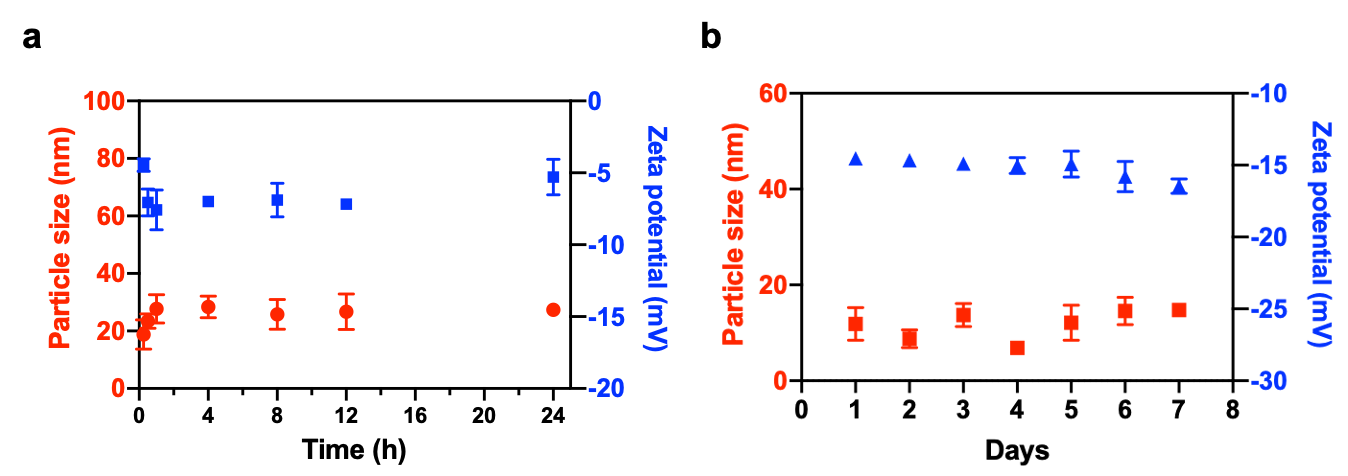
**

**Figure S1.** Colloid stability of Fe-Cur NCPs (a) in fetal bovine serum (FBS) at 37 °C for 24 h, and (b) in PBS at 4 °C for 7 days.

*
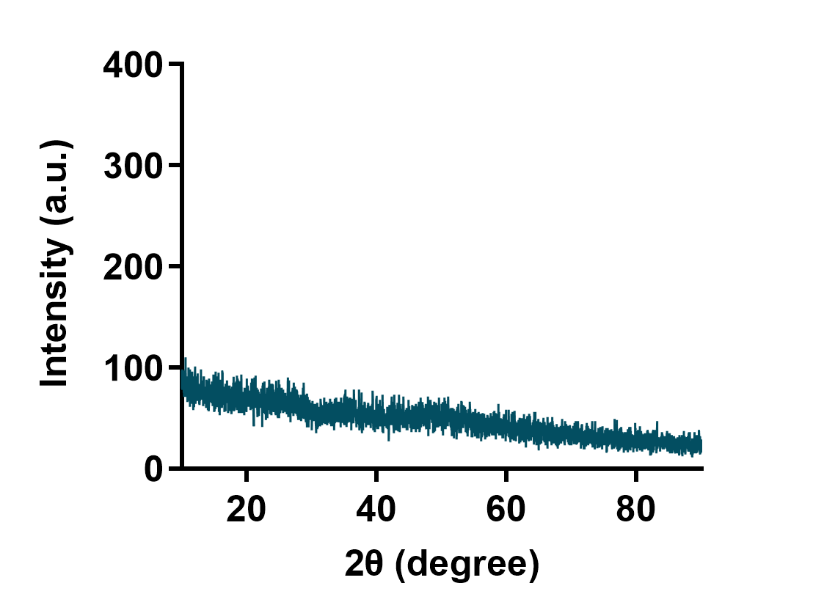
*

**Figure S2** X-ray diffraction (XRD) spectrum of Fe-Cur NCPs.


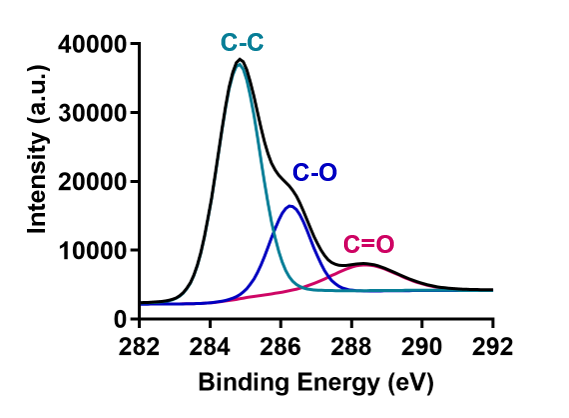


**Figure S3** X-ray photoelectron spectroscopy (XPS) spectra of Fe-Cur NCPs.

**
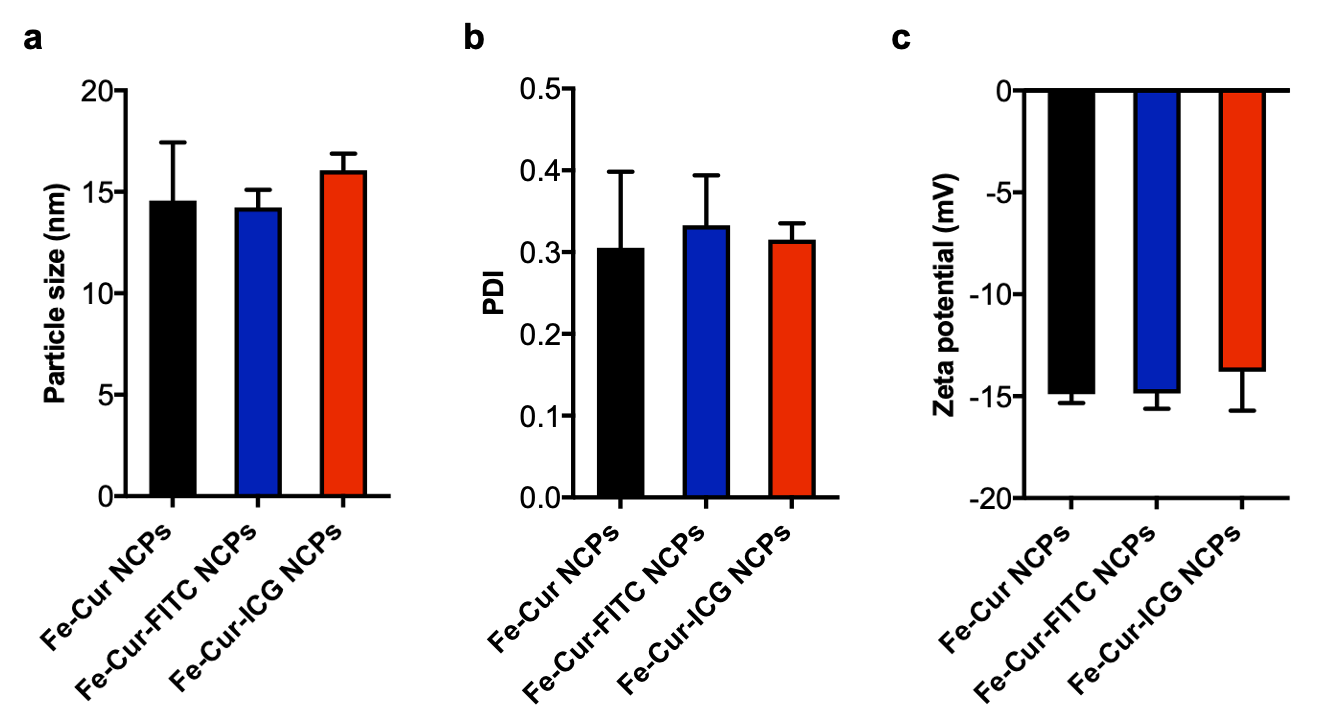
**

**Figure S4.** (a) Particle size, (b) PDI and (c) zeta potential of Fe-Cur NCPs after labeled by FITC (Fe-Cur-FITC NCPs) or ICG (Fe-Cur-ICG NCPs).


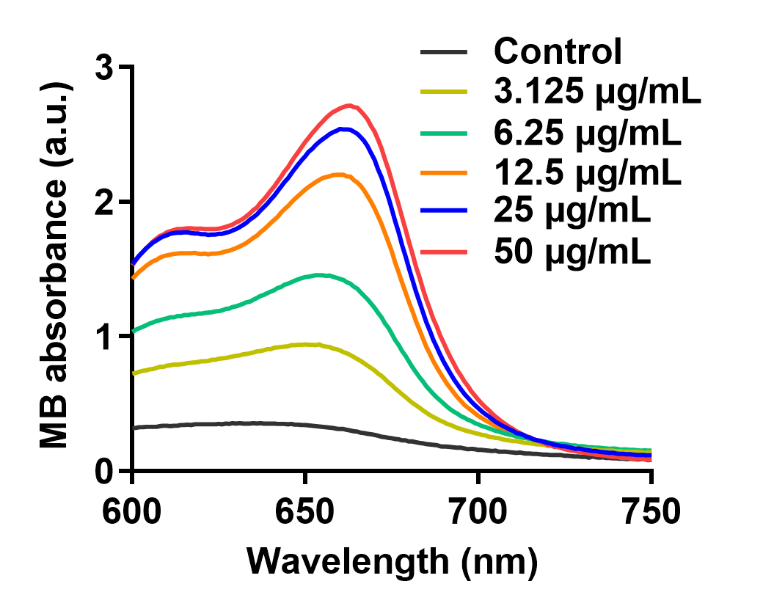


**Figure S5** Absorbance spectra of methylene blue (MB) after treatment with different concentrations of Fe-Cur NCPs.


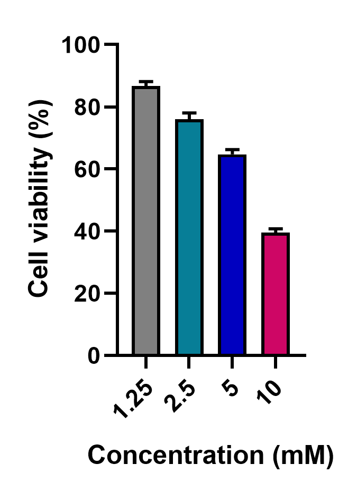


**Figure S6** Cytotoxicity after treatment with different concentrations of MβCD to bEnd.3 cells.


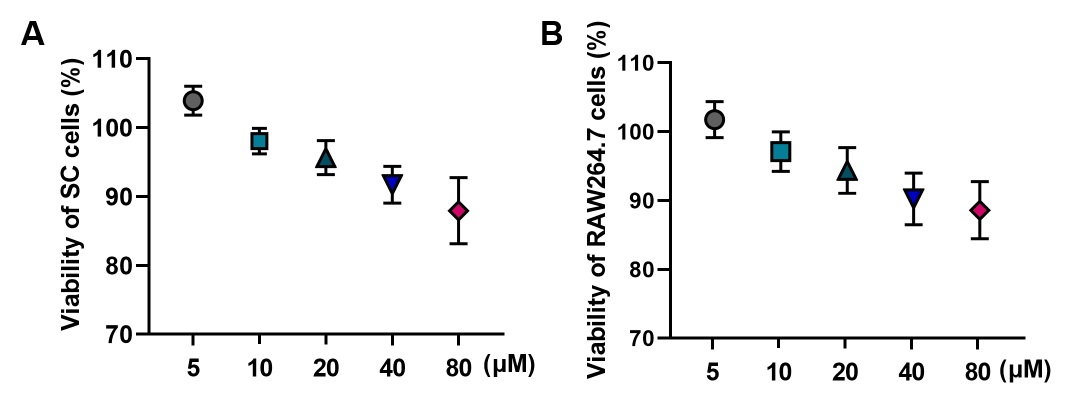


**Figure S7** Cytotoxicity analysis in RAW264.7 (A) and SC (B) cells treated with different concentrations of Fe-Cur NCPs (n=3).


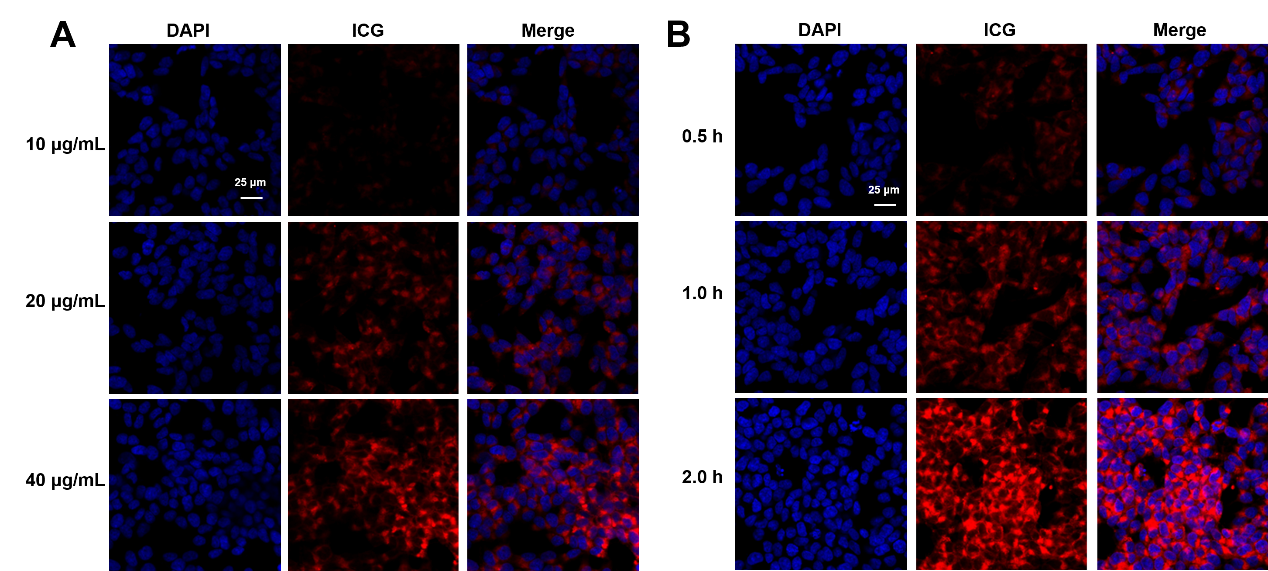


**Figure S8** Cellular uptake of Fe-Cur-ICG NCPs in SH-SY5Y cells. (A) Concentration-dependent images after incubation for 1 h. (B) Time-dependent images after incubation with 20 μg/mL Fe-Cur-ICG NCPs.


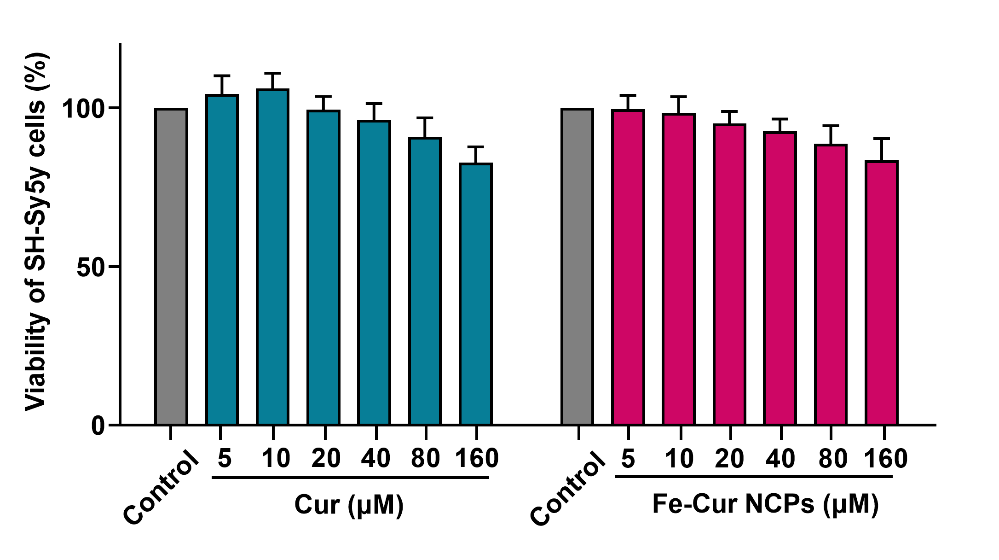


**Figure S9** Cytotoxicity analysis in SH-SY5Y cells treated with different concentrations of Cur or Fe-Cur NCPs (n=3).


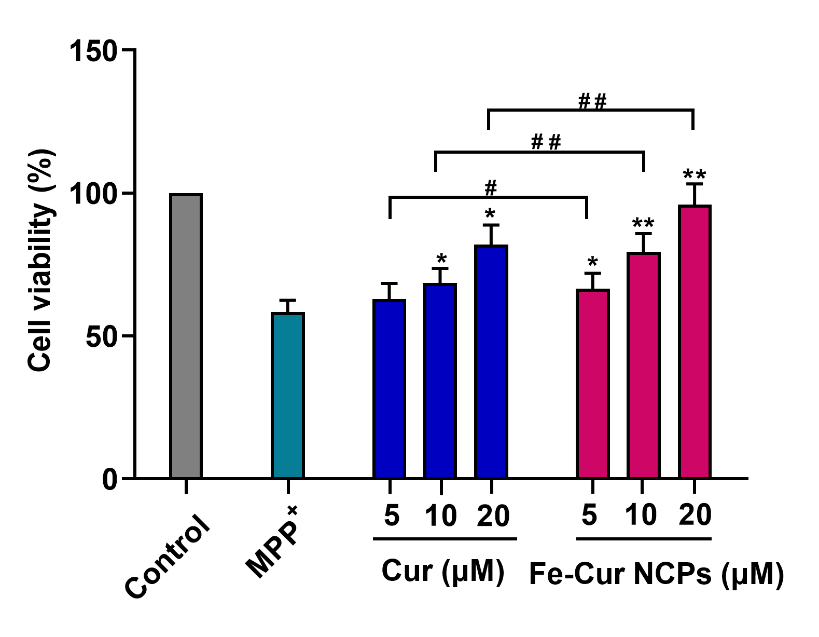


**Figure S10** Cell viability of SH-SY5Y cells treated with MPP^+^ and different concentrations of Cur or Fe-Cur NCPs. Compared with the MPTP group: **P* < 0.05 and ***P* < 0.01. Compared with the Cur group: ^#^*P* < 0.05 and ^##^*P* < 0.01 (n=3).


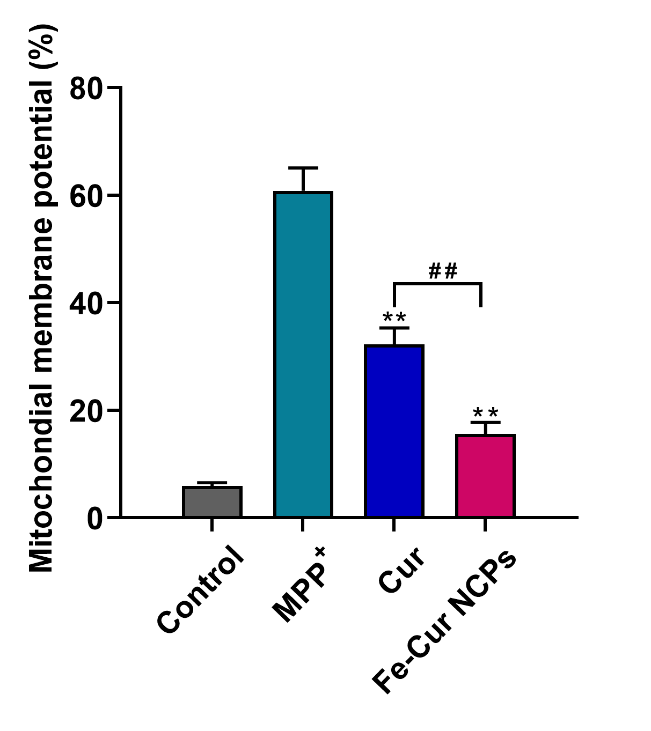


**Figure S11** The effects of Cur and Fe-Cur NCPs (both Cur concentration of 20 μM) on mitochondrial membrane potential. Compared with the MPTP group: **P* < 0.05 and ***P* < 0.01. Compared with the Cur group: ^#^*P* < 0.05 and ^##^*P* < 0.01 (n=3).


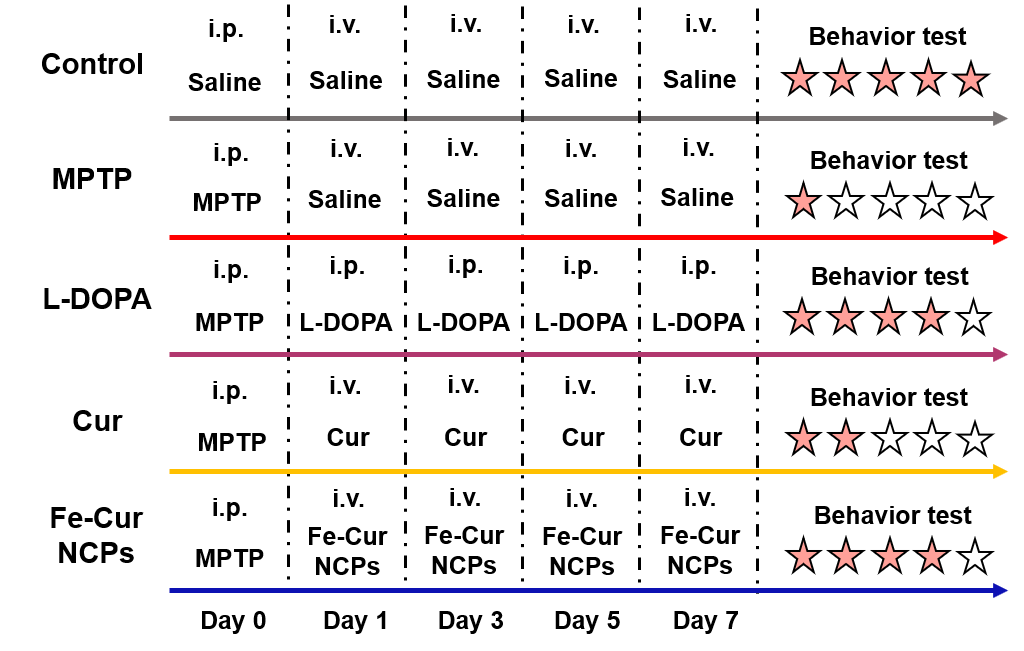


**Figure S12** Schematic illustration of pharmacodynamic study and performances.


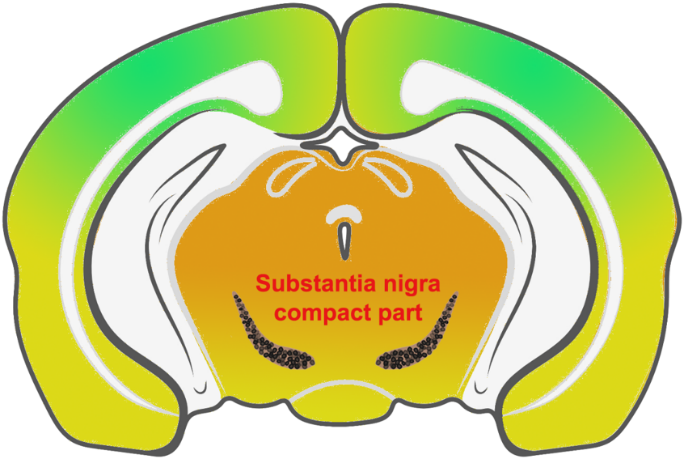


**Figure S13** Anatomical location of the substantia nigra pars compacta (SNpc).


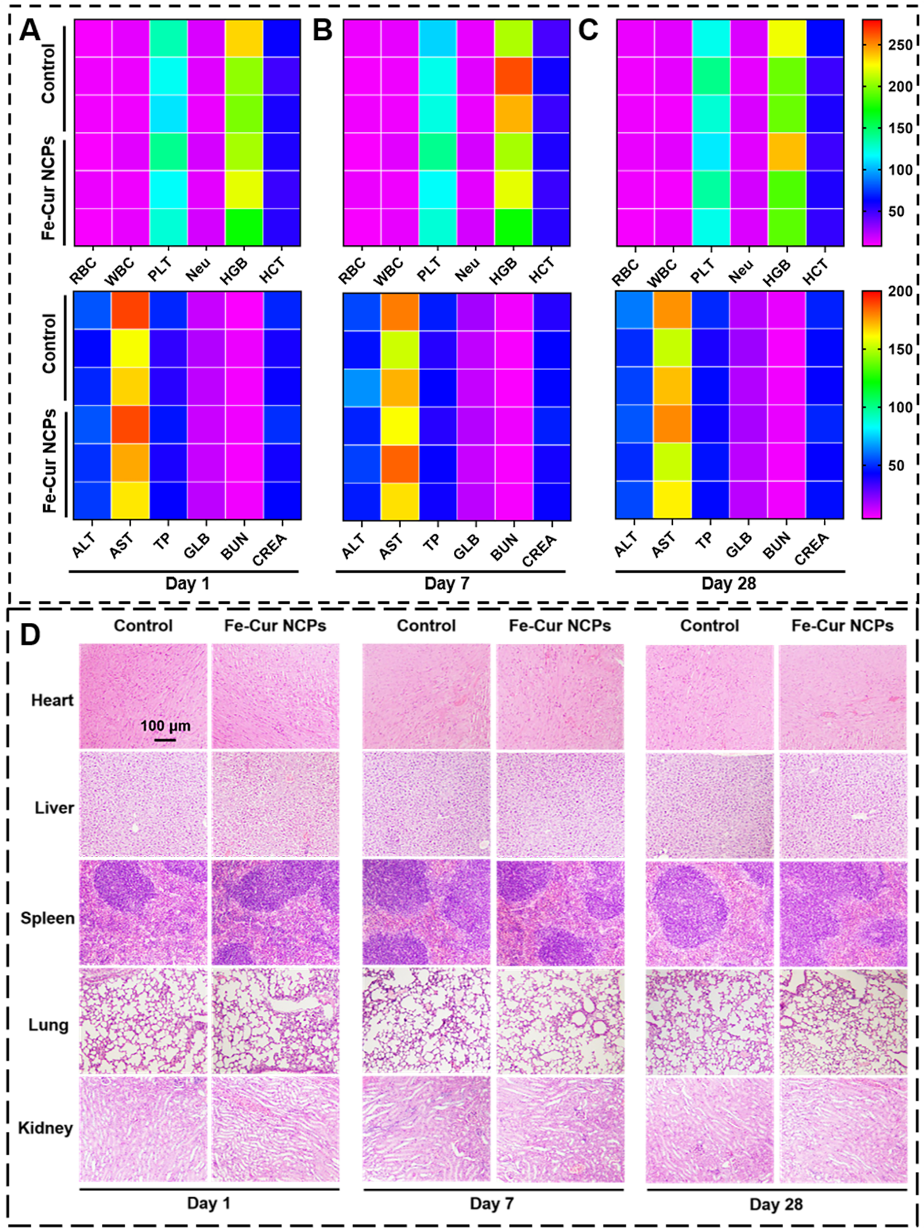


**Figure S14** ***In vivo* toxicity evaluation.** (A-C) Hematological indexes and blood biochemistry (alanine aminotransferase, ALT; aspartate aminotransferase, AST; total protein, TP; globulin, GLB; blood urea nitrogen, BUN; creatinine, CREA; Red blood cell, RBC; White blood cell, WBC; Platelet, PLT; neutrophil, Neu; Hemoglobin, HGB; hematocrit, HCT) examined in mice treated with Fe-Cur NCPs (10 mg/kg) at (A) day 1, (B) day 7, and (C) day 28 (n=3). (D) Histological examinations of the main organs (heart, liver, spleen, lung and kidney) of mice treated with Fe-Cur NCPs (10 mg/kg) at different time points using H&E staining.


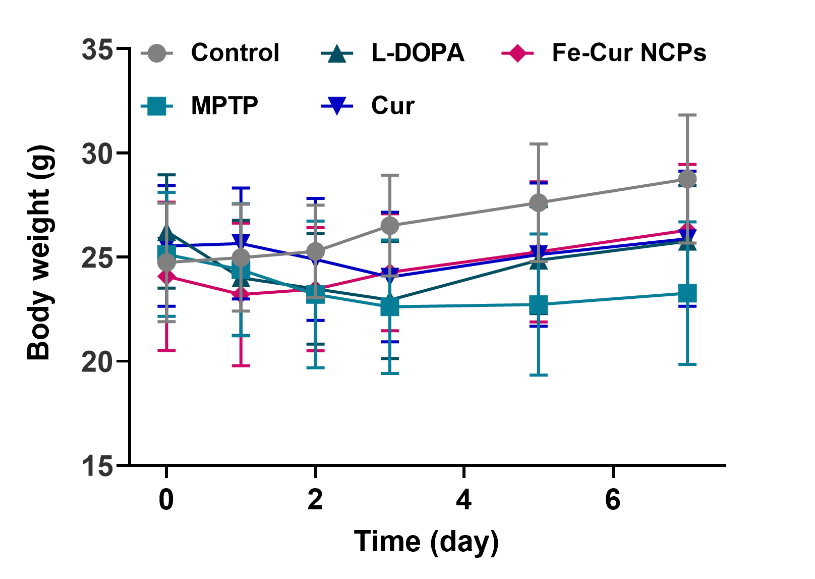


**Figure S15** Body weights of PD mice during various therapeutic treatments indicated.


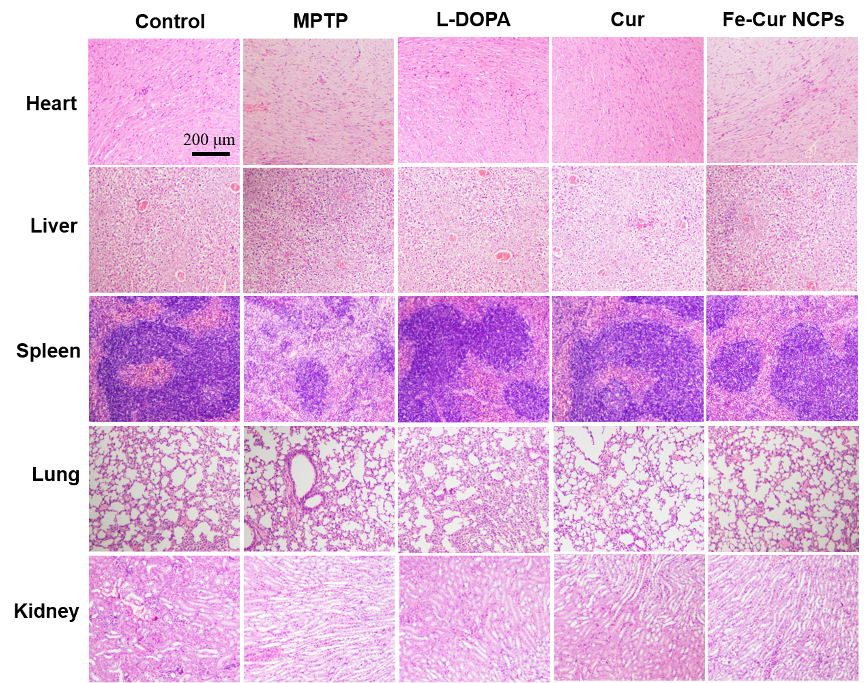


**Figure S16** H&E-stained images of the indicated organs of PD mice in different treatment groups.


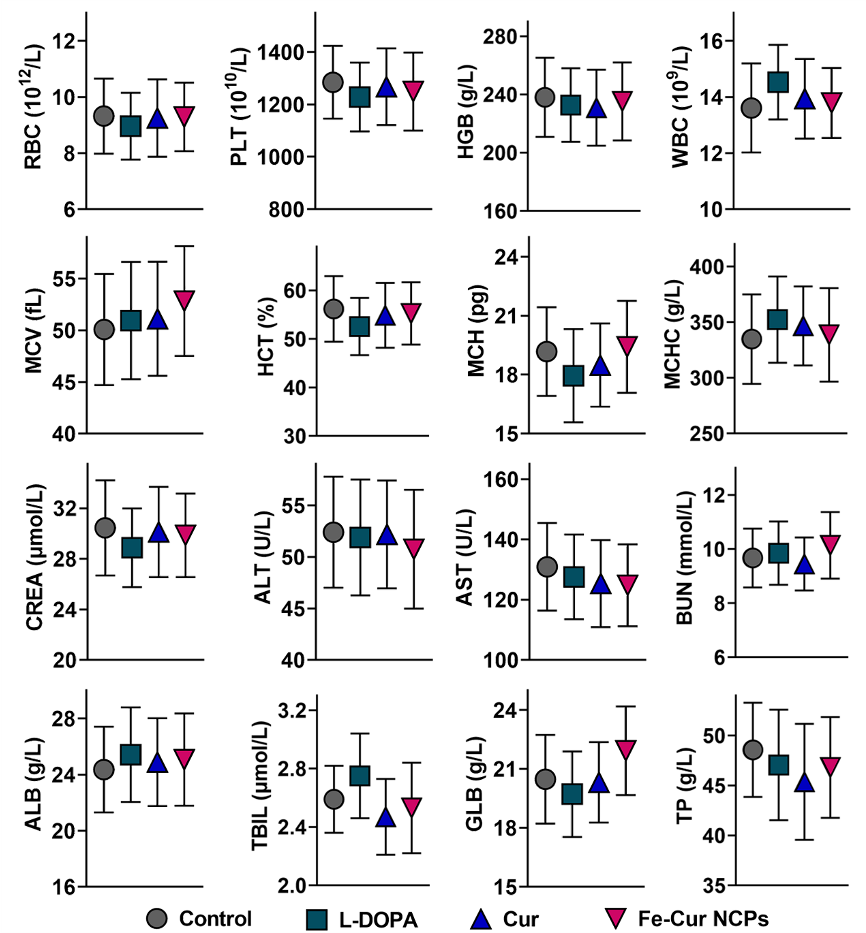


**Figure S17** Haematological parameters and blood biochemical analyses of the PD mice treated with different groups (n=6).


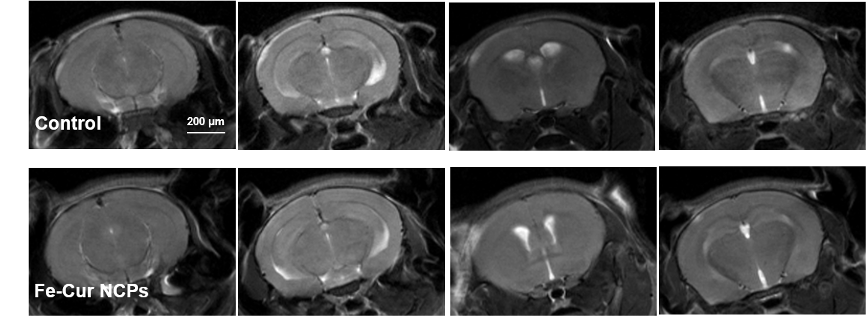


**Figure S18** MRI scans of mice brains in control and Fe-Cur NCPs groups. Scale bar: 200 μm.
